# Exploring the Spatiotemporal Evolution and Socioeconomic Determinants of PM2.5 Distribution and Its Hierarchical Management Policies in 366 Chinese Cities

**DOI:** 10.3389/fpubh.2022.843862

**Published:** 2022-03-09

**Authors:** Minli Zhu, Jinyuan Guo, Yuanyuan Zhou, Xiangyu Cheng

**Affiliations:** ^1^School of Criminal Justice, Zhongnan University of Economics and Law, Wuhan, China; ^2^School of Information and Safety Engineering, Zhongnan University of Economics and Law, Wuhan, China; ^3^The Co-innovation Center for Social Governance of Urban and Rural Communities in Hubei Province, Zhongnan University of Economics and Law, Wuhan, China

**Keywords:** air pollution, city, spatiotemporal specificity, driving factors, environmental policy

## Abstract

From 2013 to 2017, progress has been made by implementing the *Air Pollution Prevention and Control Action Plan*. Under the background of the *3 Year Action Plan to Fight Air Pollution* (2018–2020), the pollution status of PM2.5, a typical air pollutant, has been the focus of continuous attention. The spatiotemporal specificity of PM2.5 pollution in the Chinese urban atmospheric environment from 2018 to 2020 can be summarized to help conclude and evaluate the phased results of the battle against air pollution, and further, contemplate the governance measures during the period of the 14th Five-Year Plan (2021–2025). Based on PM2.5 data from 2018 to 2020 and taking 366 cities across China as research objects, this study found that PM2.5 pollution has improved year by year from 2018 to 2020, and that the heavily polluted areas were southwest Xinjiang and North China. The number of cities with a PM2.5 concentration in the range of 25–35 μg/m^3^ increased from 34 in 2018 to 86 in 2019 and 99 in 2020. Moreover, the spatial variation of the PM2.5 gravity center was not significant. Concretely, PM2.5 pollution in 2018 was more serious in the first and fourth quarters, and the shift of the pollution's gravity center from the first quarter to the fourth quarter was small. Global autocorrelation indicated that the space was positively correlated and had strong spatial aggregation. Local Moran's I and Local Geti's G were applied to identify hotspots with a high degree of aggregation. Integrating national population density, hotspots were classified into four areas: the Beijing–Tianjin–Hebei region, the Fenwei Plain, the Yangtze River Delta, and the surrounding areas were selected as the key hotspots for further geographic weighted regression analysis in 2018. The influence degree of each factor on the average annual PM2.5 concentration declined in the following order: (1) the proportion of secondary industry in the GDP, (2) the ownership of civilian vehicles, (3) the annual grain planting area, (4) the annual average population, (5) the urban construction land area, (6) the green space area, and (7) the per capita GDP. Finally, combined with the spatiotemporal distribution of PM2.5, specific suggestions were provided for the classified key hotspots (Areas A, B, and C), to provide preliminary ideas and countermeasures for PM2.5 control in deep-water areas in the 14th Five-Year Plan.

## Introduction

Atmospheric particulate matter <2.5 μm in diameter (PM2.5) pollution has become a worldwide challenge, especially for some developing countries (1, 2). Chinese urban areas have also experienced relatively high air pollution (3). PM2.5 is considered to be one of the most harmful pollutants (4), often coming from coal combustion, industrial activities, vehicle emissions, and so on (5), and it absorbs toxins, such as organic toxic components and heavy metals (6, 7). PM2.5 can increase mortality because it can cause a variety of diseases (8–11) and result in economic losses (12).

Air quality has sparked widespread social debate and China is gradually strengthening its monitoring of PM2.5, which was included in the Chinese conventional key monitoring indicators in 2012. In 2013, there were 612 PM2.5 monitoring stations in China. Since 2015, China has founded a monitoring network covering big cities (13), which has increased the number of monitoring stations to 1,602 as of February 2018. Based on abundant site monitoring data and experiments, some researchers have studied the source, chemical composition, and spatiotemporal characteristics of PM2.5 pollution and its impact on human health (14–16). For example, the spatiotemporal correlation of particulate matter in the Beijing–Tianjin–Hebei region (17) and the sequence pattern of PM2.5 pollution in some regions of China (18) have been studied. In response to the short-term and long-term challenges of PM2.5, the Chinese government has taken several measures, including the *Air Pollution Prevention and Control Action Plan* (2013–2017) and the *3-year Action Plan to Fight Air Pollution* (2018–2020). Since 2013, China has launched a series of initiatives aimed at PM2.5 pollution control, with remarkable achievements in the areas of coal-fired pollution and mobile source pollution control, and the air quality has advanced considerably. Compared with PM2.5 pollution in 2013, the annual average of PM2.5 in the Beijing–Tianjin–Hebei region, the Yangtze River Delta, and the Pearl River Delta declined by 40, 34, and 28%, respectively, in 2017 (19). However, many regions and cities in China are still facing the urgent need to solve PM2.5 pollution, so the work toward improving Chinese air quality still faces great challenges. Therefore, in the face of the goal of a 10% decrease in PM2.5 pollution during the period of the 14th Five-Year Plan (2021–2025), the spatiotemporal specificity of urban PM2.5 pollution in China from 2018 to 2020 can be summarized to help conclude the phased results of the battle against air pollution, and then identify the further challenges and corresponding targeted policies, to promote China's active response to the continuous enhancement of air quality.

In the formulation of different management measures related to PM2.5, it is necessary to discuss the characteristics of different sources, to achieve an accurate and effective control effect. The research trend in recent years shows that scholars are more and more interested in using a spatial econometric analysis method to track the geographical changes of PM2.5 and its socioeconomic driving factors. Taking China as an example, it was found that the spatial distribution of PM2.5 concentration showed an increasing trend from the east to the west (20, 21), and the spatial autocorrelation and clustering characteristics of 338 cities in China were remarkable (22). The urban PM2.5 in the Bohai region of China showed high spatial variation and agglomeration characteristics (23), and the haze pollution in China showed time path dependence and a spatial spillover effect (24). A large amount of literature has studied the socioeconomic driving factors of PM2.5 in a broader sense, indicating that population agglomeration (25, 26), economic growth (27), industrial structure (28), energy structure and efficiency, land-use type (29), and other factors may be the main driving factors of PM2.5 concentration. The spatial scales of urban air quality control were macro (national level), medium (urban level), and micro (specific location) (30). Compared with the medium and micro scales, few researchers have attempted to conduct hierarchical multi-scale studies from a systematic country–region–city perspective for policymaking in the period of China's 14th Five-Year Plan (31). Furthermore, local governments should not adopt a one-size-fits-all policy, but rather establish hierarchical policies according to different social and economic development conditions and needs of Chinese cities (32, 33). In addition, effective communication and cooperation among relevant cities were also important (34). Urban air pollution has the complexity of multi-source, multi-scale, and cross-regional distribution. These problems cannot be solved only by pollution control in one city, and cooperation is needed to reduce transboundary pollutants, such as PM2.5 (35). Therefore, from a country–region–city perspective, identifying the regional pattern of thermal pollution driven by a variety of socio-economic forces and formulating targeted joint management strategies for each regional pattern are of great significance for efficient and fair PM2.5 pollution control in the future (36). Undertaking the *3-Year Action Plan to Fight Air Pollution* and standing at the starting point of the 14th Five-Year Plan, this study analyzed the spatiotemporal distribution and socioeconomic driving factors of PM2.5 pollution from 2018 to 2020, to provide in-depth, hierarchical, and collaborative management countermeasures for the period of the 14th Five-Year Plan.

The objectives of this study are as follows: (1) to characterize the geographical center of PM2.5 pollution and use Global Moran's I method for global autocorrelation analysis, to investigate the spatiotemporal distribution characteristics of PM2.5 from 2018 to 2020; (2) to identify the socioeconomic driving factors in the identified key hotspots by geographic weighted regression (GWR) analysis; (3) to classify the key hotspots by integrating the local autocorrelation analysis of pollution hotspots and the population density features, and to comprehensively provide feasible and targeted control measures, to provide evidence for the air pollution control of Chinese cities.

## Materials and Methods

### Data Collection

PM2.5 data came from the ground monitoring data of 366 cities in China from 2018 to 2020 (excluding Hong Kong, Macao, and Taiwan due to the lack of available data; http://106.37.208.233:20035/). Most of the 366 cities have established national air quality monitoring stations, and most of the stations are in urban areas. Each monitoring station has a monitoring system according to *Technical Specifications for Installation and Acceptance of Ambient Air Quality Continuous Automated Monitoring System for PM10 and PM2.5* (HJ655-2013) to measure the concentration of fine particles per hour and per day. According to the ground monitoring data provided by the air quality monitoring station, first, some outliers (such as some hourly PM2.5 concentrations that were <0 and missing values) were removed. Second, the daily average of PM2.5 is calculated only when the effective hourly data of the day is more than or equal to 20 h (37). When calculating the monthly average, the effective PM2.5 days per month must be more than or equal to 27 [at least 25 in February; (38)]. Finally, the daily, monthly, seasonal, and annual mean (AM) of the PM2.5 concentrations of the monitoring points were obtained according to the arithmetic average method.

As for economic factor data, based on the Delphi method and available studies (39), a total of 18 indicators were chosen, which were the annual average population (10,000 people), urban construction land area (square kilometers), GDP (the current price; 10,000 Chinese yuan), per capita GDP (Chinese yuan), GDP growth rate (%), the proportion of the primary industry in the GDP, the proportion of the secondary industry in the GDP, the proportion of the tertiary industry in the GDP, the employees (people) of the primary industry (agriculture, forestry, animal husbandry, and fishery), the employees (people) of the secondary industry, the employees (people) of the tertiary industry, the proportion of the employees of the primary industry, the proportion of the employees of the secondary industry, the proportion of the employees of the tertiary industry, the green space area (hectare), the ownership of civilian vehicles (10,000 vehicles), and the annual grain planting area (10,000 mu). The data were from the *China City Statistical Yearbook* (http://www.stats.gov.cn/tjsj/tjcbw/), provincial statistical yearbooks (http://www.stats.gov.cn/tjgz/wzlj/dftjwz/), and some urban statistical bulletins.

### Data Analysis

#### Gravity Model

This study revealed the spatial migration process of PM2.5 by the concept and calculation method of the gravity center in physics. Moreover, the study also characterized the geographical center of PM2.5 pollution by the gravity model. The X and Y coordinates of the PM2.5 pollution center in China are defined as:


(1) $$\begin{array}{l} \bar{X}=\overset{n}{\underset{i=1}{\sum }}X_{i}\times S_{i}\times \frac{W_{i}}{\overset{n}{\underset{i=1}{\sum }}S_{i}}\times W_{i} \end{array}$$

(2) $$\begin{array}{l} \bar{Y}=\overset{n}{\underset{i=1}{\sum }}Y_{i}\times S_{i}\times \frac{W_{i}}{\overset{n}{\underset{i=1}{\sum }}S_{i}}\times W_{i} \end{array}$$

where $\bar{X}$ is the longitude of the gravity of the PM2.5 pollution center; the latitude of the gravity of the PM2.5 pollution center is expressed by $\bar{Y}$; the number of grids in the research scope is represented by *n*; the grid number is represented by *i*; *X*_*i*_ and *Y*_*i*_ represent the longitude and latitude of the geometric center of the grid whose number is *i*, respectively; *S*_*i*_ represents the area of the grid *i*; and *W*_*i*_ represents the AM of the PM2.5 of the grid *i* (μg/m^3^).

#### Spatial Autocorrelation Analysis Method

Spatial correlation refers to the correlation of the same variable at different locations (40). A variable in a certain position increases or decreases simultaneously with the same variable in its adjacent position, which is called spatial positive correlation. When one increases and one decreases, it is a spatial negative correlation. Spatial autocorrelation analysis describes the potential interdependence of some variables in adjacent spatial units and explores the spatial aggregation mode of factors, which can reveal the potential trend of environmental pollution in recent years (41, 42). According to the size of the analysis space, spatial autocorrelation can be divided into global spatial autocorrelation and local spatial autocorrelation (43), which are listed in the Supporting Information.

#### Geographic Weighted Regression Model and the Variance Inflation Factor

The change in PM2.5 may be related to industrial activities and human life. Considering the potential multicollinearity between these socio-economic factors, the variance inflation factor (VIF) is usually used for screening. Independent variables with VIF values of more than 10 should be removed before using GWR. The *p*-value is considered an indicator to determine the reliability of Moran's I. When the *p*-value of Moran's I is lower than 0.05, spatial econometrics and GWR can be applied. Otherwise, the application of spatial econometrics or GWR is unreasonable. Therefore, the GWR model is used to calculate the coefficients (R) and local regression coefficients for each city in the study area, and these coefficients are then mapped to show spatial variability.

The GWR model is shown as follows:


(3) $$\begin{array}{l} y_{i}=\beta_{0}\left(u_{i},v_{i}\right)+\underset{k}{\sum }\beta_{k}\left(u_{i},v_{i}\right)x_{ik}+\varepsilon_{i} \end{array}$$

where *y*_*i*_ represents the concentration of air pollutants in city *i*, μg/m^3^; *(u*_*i*_*, v*_*i*_*)* represents the geographical location of city *i*; β_0_*(u*_*i*_*, v*_*i*_*)* represents the concept of city *i*; β_*k*_ *(u*_*i*_*, v*_*i*_*)* represents the local regression coefficient of meteorological factor *k*; *x*_*ik*_ represents the value of the corresponding meteorological factor in city *i*; and ε_*i*_ represents the residual of city *i*. The local regression coefficient is calculated as follows:


(4) $$\begin{array}{l} \beta \left(u_{i},v_{i}\right)={\left(X^{T}W\left(u_{i},v_{i}\right)X\right)}^{-1}X^{T}W\left(u_{i},v_{i}\right)Y \end{array}$$

(5) $$\begin{array}{l} X=\left[\begin{array}{c} 1,x_{11},\ldots ,x_{ik}\\ 1,x_{21},\ldots ,x_{2k}\\ \ldots \\ 1,x_{n1},\ldots ,x_{nk} \end{array}\right] \end{array}$$

(6) $$\begin{array}{l} W\left(u_{i},v_{i}\right)=\left[\begin{array}{c} w_{1}\left(u_{i},v_{i}\right),0,\ldots ,0\\ 0,w_{2}\left(u_{i},v_{i}\right),\ldots ,0\\ \ldots \\ 0,0,\ldots ,w_{n}\left(u_{i},v_{i}\right) \end{array}\right] \end{array}$$

where β*(u*_*i*_*, v*_*i*_*)* represents the local regression coefficients in cities; *x* represents the meteorological factor matrix; *x*^*T*^ is the transposition matrix of *X*; *w(u*_*i*_*, v*_*i*_*)* is the spatial weight matrix; *y* means the matrix of air pollutants; *k* means the number of meteorological factors; and *n* represents the number of samples.

## Results and Discussion

### Spatiotemporal Distribution Characteristics of PM2.5 From 2018 to 2020

#### Temporal Distribution Characteristics From 2018 to 2020

According to the provisions of GB3095-2012 (Supplementary Table 1), limiting values for the daily average of PM2.5 concentrations are 15 for Level I and 35 for Level II, and limiting values for the hourly average are 35 for Level I and 75 for Level II. To analyze the pollution level clearly, the PM2.5 content was further divided into five intervals: 15–25, 25–35, 35–55, 55–75, and >75 μg/m^3^. The number of Chinese cities in different PM2.5 content intervals was counted, as shown in Supplementary Figure 1 and Supplementary Table 2. In 2018, the number of cities with an annual PM2.5 concentration in the range of 15–25 μg/m^3^ was the smallest, which was zero. As the years progressed, the number of cities in the range of the annual PM2.5 concentration gradually increased. The number of cities with a PM2.5 concentration in the range of 25–35 μg/m^3^ increased from 34 in 2018 to 86 in 2019 and 99 in 2020. The number of cities with a PM2.5 concentration in the range of 35–55 μg/m^3^ also showed an increasing trend. The average annual concentration of PM2.5 in the range of 55–75 μg/m^3^ showed a significant decreasing trend, from 103 in 2018 to 61 in 2019 and 14 in 2020. This indicated that the air quality was getting better year by year. In the range of the annual PM2.5 concentration above 75 μg/m^3^, the number of cities was seven in 2018, one in 2019, and two in 2020, indicating that the overall trend of pollution was decreasing. Through the above analysis, PM2.5 pollution has improved year by year from 2018 to 2020, thanks to policy support; policies have effectively promoted the improvement of air quality.

#### Spatial Distribution Characteristics

##### Gravity Model Analysis

According to the statistical analysis of the geographical locations of the national PM2.5 pollution centers in 2018–2020 (Supplementary Figure 2), the pollution center was located in Anqing in 2018, moved northwestward to Huanggang in 2019, and moved southwestward in 2020, but it was still located in Huanggang (Hubei province). A detailed analysis is listed in the Supporting Information.

##### Macro Analysis

**Figure 1:**
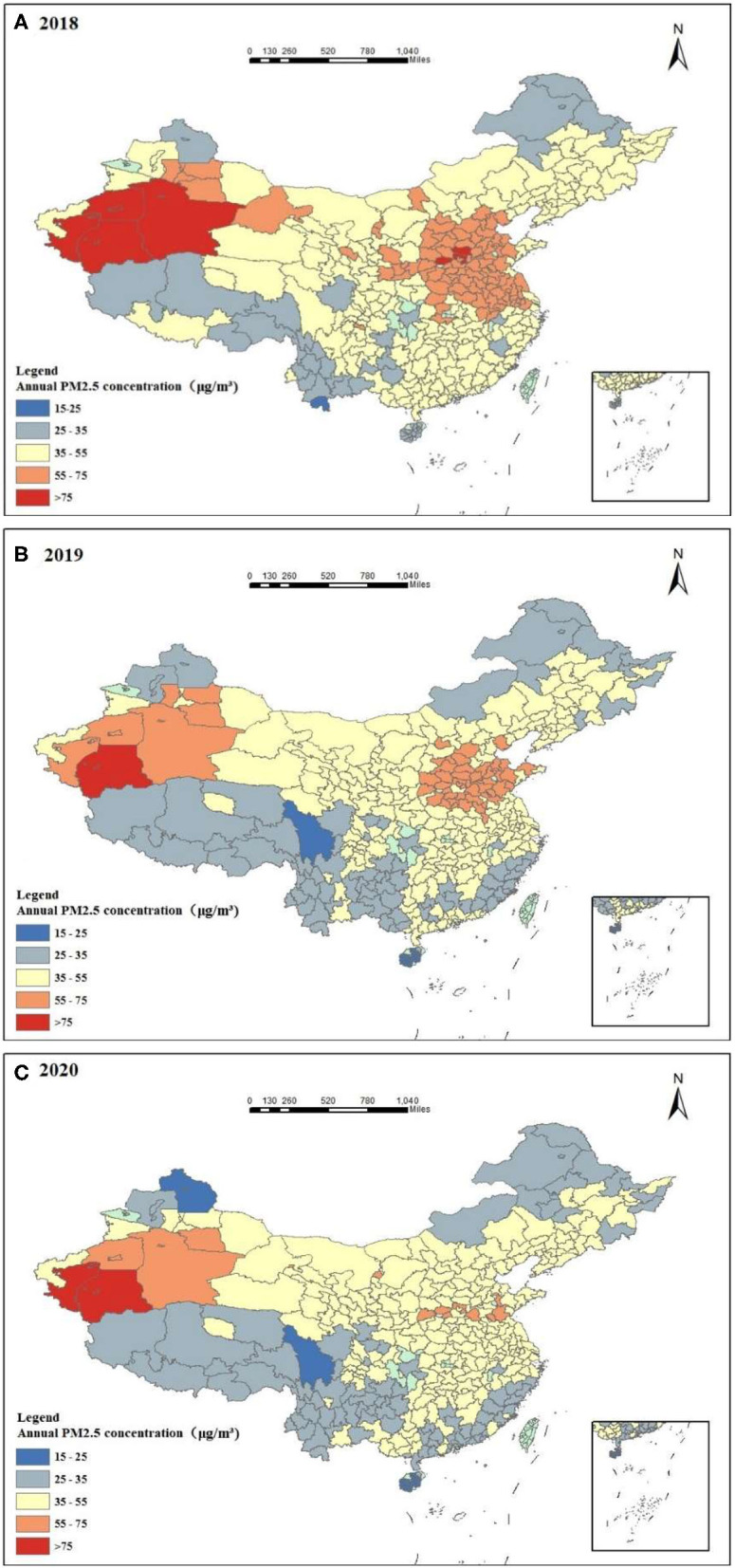
Annual PM2.5 concentration distribution in 2018 **(A)**, 2019 **(B)**, and 2020 **(C)**.

The AM distribution map of PM2.5 from 2018 to 2020 (Figure 1) shows that the heavily polluted areas were southwest Xinjiang and North China. The pollution situation in southern China and the Qinghai–Tibet Plateau was relatively light. The factors that caused the above phenomenon may be different industrial structures, a high proportion of the heavy industry, and a different population. The serious pollution in western Xinjiang was mainly caused by an arid climate and low rainfall-related meteorological conditions on the western edge of the Taklamakan Desert; thus, it was less affected by human activities.

### Spatiotemporal Distribution Characteristics of PM2.5 in 2018

According to the annual evolution trend, PM2.5 pollution in 2018–2020 was improving year by year. On the whole, there were few areas with serious pollution, and the spatial distribution also showed certain regularity. However, due to the impact of the pandemic from 2019 to 2020, there were some uncertain factors. Therefore, this study selected the PM2.5 pollution status of Chinese cities in 2018 as the main research object for further specific analysis.

#### Quarterly Characteristics

**Figure 2:**
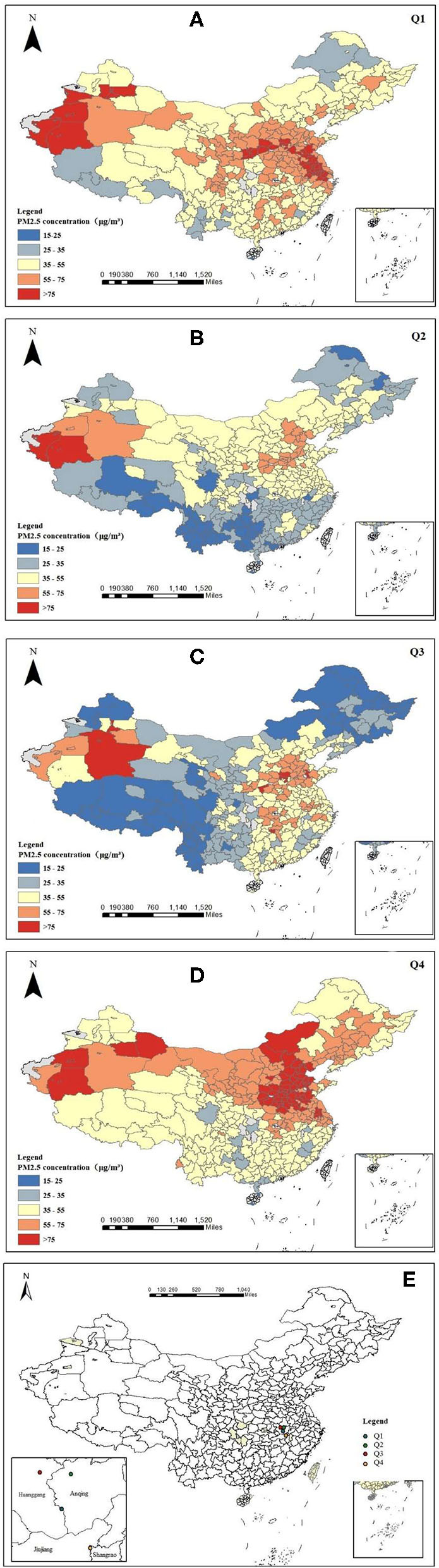
Quarterly distribution map of PM2.5 in 2018 **(A–D)** and the distribution map of the pollution gravity center in 2018 **(E)**.

Figures 2A–D shows that PM2.5 pollution was more serious in the first and fourth quarters of 2018, followed by the second and third quarters. Areas with a PM2.5 concentration of more than 75 μg/m^3^ in the first quarter were mainly distributed in Xinjiang, such as Kashgar, Hotan, Turpan, and Urumqi, as well as Baoding, Weifang, Suihua, Shijiazhuang, and Zaozhuang. There are two main sources of PM2.5. In terms of climate, the rainfall in the first quarter was less than in other quarters. Thanks to warmer weather and increased rain, fine particles can be washed away, and the PM2.5 concentration then goes down in the second quarter. In the third quarter, only the PM2.5 concentration near Hotan in Xinjiang was >75 μg/m^3^, and the pollution in the Beijing–Tianjin–Hebei region was serious. In the fourth quarter, the weather turned cold, the northern region began to heat up, and the pollution in northern China was serious.

The geographical location of the PM2.5 pollution center in the four quarters of 2018 was statistically analyzed. It can be seen from Figure 2E that the pollution center in the first quarter was located at the boundary between Huanggang and Anqing. In the second quarter, it moved to the northeast and was located in Anqing. In the third quarter, it moved westward and was located in Huanggang. In the fourth quarter, it moved southeastward at a large distance and was located at the boundary between Jiujiang and Shangrao. From a macro point of view, the shift of the pollution gravity center from the first quarter to the fourth quarter was small, so it can be seen that the PM2.5 pollution in different quarters across the country did not show significant regional differences in terms of increases and decreases.

#### Spatial Autocorrelation Analysis

##### Global Autocorrelation

According to the analysis results (Supplementary Table 3), Global Moran's I was 2.304041, which was positive, and it indicated that the space was positively correlated and had strong spatial aggregation. The *P*-value was <0.05, so the data had analytical significance. The *Z*-value was 73.941729, much more than 1.96, which proved that Global Moran's I meets the test conditions. There was a significant spatial agglomeration phenomenon in the AM distribution of PM2.5 in Chinese cities in 2018.

##### Local Autocorrelation

Local autocorrelation analysis was used to study the distribution of PM2.5 in local areas. Local Moran's I (GeoDa mapping) and Local Geti's G (ArcGIS mapping) were used, and the clustering maps of the local indicators of spatial association were obtained. Zhang and Zhang (44) compared the differences between Moran's I and Geti's G in detecting spatial aggregation by designing a large number of simulation calculations and concluded that Geti's G was better at showing the local autocorrelation. These methods were compared and a more suitable local autocorrelation analysis method for further analysis was selected.

**Figure 3:**
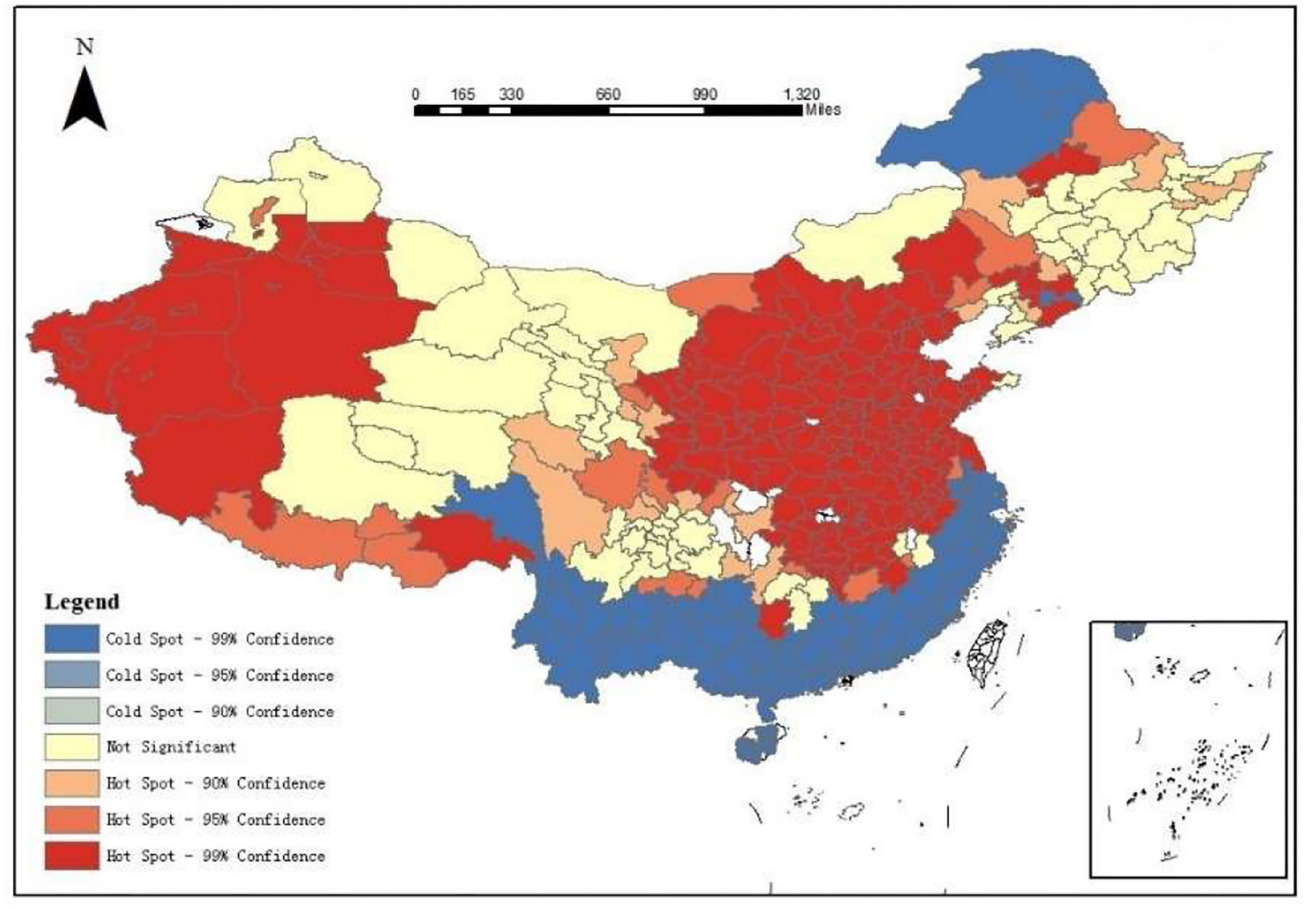
Local Geti's G.

According to Figure 3 and Supplementary Figure 3, the results obtained by Local Moran's I and Local Geti's G were roughly similar, but there were still differences. The comparison showed that Local Geti's G could accurately detect the aggregation area, whereas Local Moran's I could roughly detect the center of the aggregation area, but the recognition deviation of the aggregation range was large, and the detection range was less than the actual range (Figures 1, 2). Therefore, Local Geti's G was selected to reflect the local autocorrelation. Figure 3 shows that hotspots with a high degree of aggregation were mainly distributed in Kashgar, Hotan, Turpan, and other regions of Xinjiang, the Fenwei Plain, the Yangtze River Delta, the Beijing–Tianjin–Hebei region, and others. Cold spots were mainly distributed in coastal cities in southern China, such as Wenzhou, Longyan, and Lishui. The local aggregation degree of hotspots was high, and most of them were located in inland areas, which was not conducive to the diffusion of pollutants, such as Shaanxi and Shanxi. Due to factors, such as wind and sand in Xinjiang, the pollution situation was grim. Shandong, Hebei, and other regions had a high degree of aggregation. A joint prevention and control mechanism can be adopted in hotspots to achieve an optimal control effect.

#### Population Density

**Figure 4:**
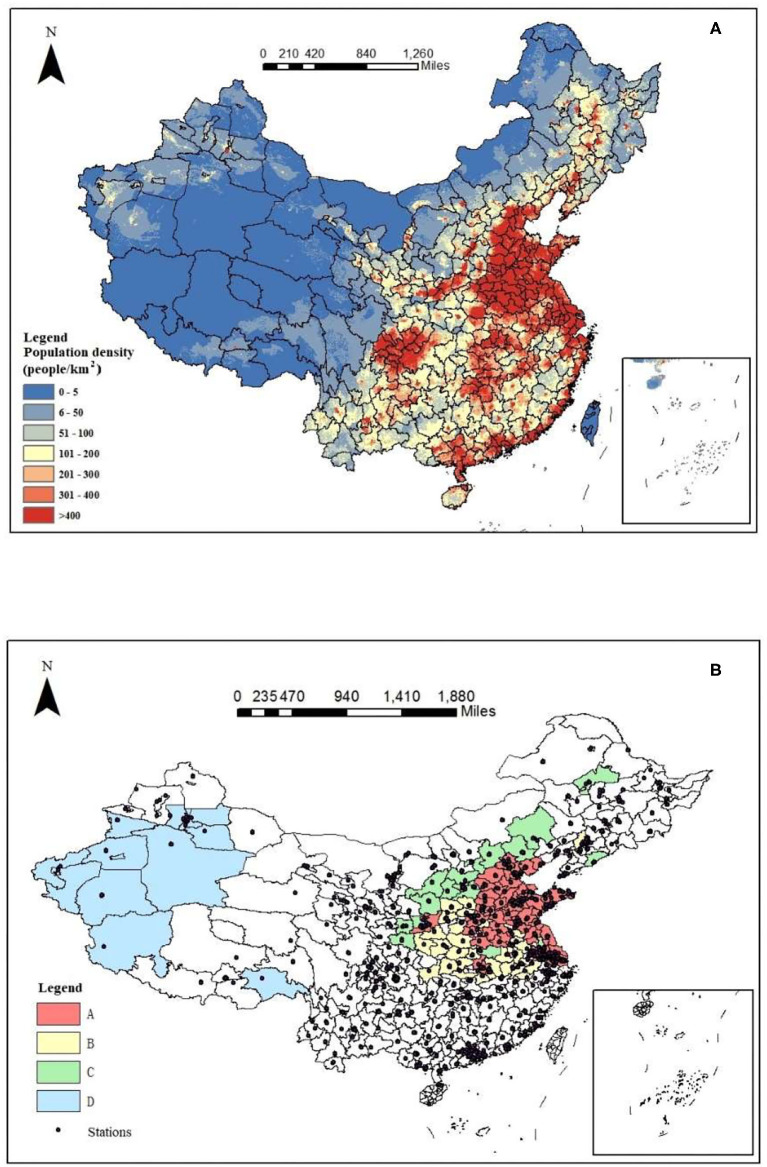
Population density map **(A)** and the distribution of hotspots and monitoring sites **(B)**.

Human activity is one of the important factors affecting PM2.5 (45). Frequent human activities will increase the concentration of PM2.5 in the atmospheric environment. Population density refers to the number of people living in a unit area, which can accurately represent the population density of a region. Based on the data of the 2010 national population census, the distribution of national population density is shown in Figure 4A. From Figure 4A, it can be seen that the population density in China showed the law of “high in the east and low in the west.” The population density was taken as one of the influencing factors in this study. Compared with the hotspots in Figure 4B, different levels of regions were divided more scientifically.

The population density level could be divided into gathering area, transition area, less sparse area, and severely sparse area, and the corresponding population density of each area is 400, 200–400, 50–200, and <50 people/km^2^. The hotspots with a population density of more than 400 people/km^2^ were considered Area A, 200–400 people/km^2^ were considered Area B, 50–200 people/km^2^ were considered Area C, and the hotspots with a population density of fewer than 50 people/km^2^ were considered Area D. The results are shown in Figure 4B. According to the distribution of monitoring sites, there were few monitoring sites in the western regions of China, and it is particularly necessary to set the classification of areas A, B, C, and D. Due to the small number of sites and the low density of the potentially exposed population in Area D, no further analysis was carried out. Due to the natural environmental conditions, such as wind and sand in Area D, the air conditions were poor. More afforestation should be carried out to increase the coverage of green plants and improve the state of pollution. For Areas A, B, and C, further driving factor analyses were carried out to develop targeted policies.

### Analysis of the Socioeconomic Driving Factors

Combined with the population density map and the distribution of hotspots, through local autocorrelation analysis, the Beijing–Tianjin–Hebei region, the Fenwei Plain, the Yangtze River Delta, and the surrounding areas were selected as the key hotspots for GWR analysis.

#### Factor Selection and the VIF Test

A total of 18 indicators were chosen as independent variables in this study. The average annual concentration of PM2.5 (μg/m^3^) was analyzed as a dependent variable. Before the GWR analysis, the VIF test should be preprocessed first to remove the indicators with VIF values of more than 10 to avoid collinearity affecting the experimental results. Then, the remaining usable indicators were the annual average population (10,000 people), urban construction land area (square kilometers), per capita GDP (Chinese yuan), GDP growth rate (%), the proportion of the primary industry in the GDP, the proportion of the secondary industry in the GDP, the employees (people) of the primary industry (agriculture, forestry, animal husbandry, and fishery), the employees (people) of the secondary industry, the employees (people) of the tertiary industry, the proportion of the employees of the primary industry, the proportion of the employees of the secondary industry, the proportion of the employees of the tertiary industry, the green space area (hectare), the ownership of civilian vehicles (10,000 vehicles), and the annual grain planting area (10,000 mu).

#### GWR Analysis

**Figure 5:**
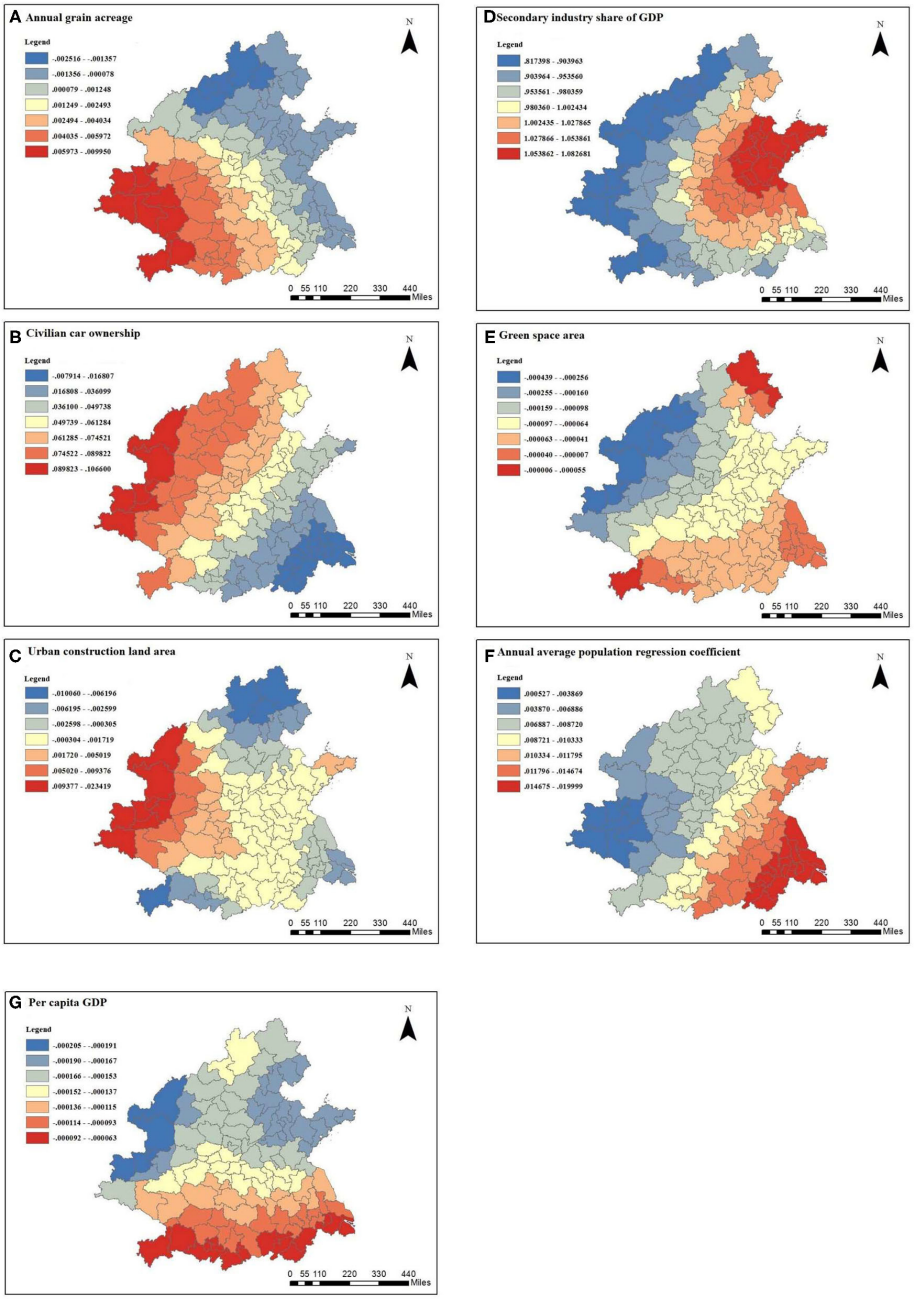
Spatial distribution of the regression coefficients of the parameters in the GWR model: **(A)** annual grain acreage, **(B)** civilian car ownership, **(C)** urban construction land area, **(D)** secondary industry share of GDP, **(E)** green space area, **(F)** annual average population regression coefficient, and **(G)** per capita GDP.

After excluding the local and global collinearity, the remaining seven factors were effective, which were the following: the annual average population (10,000 people), urban construction land area (square kilometers), the per capita GDP (Chinese yuan), the proportion of the secondary industry in the GDP, the green space area (hectares), the ownership of civilian vehicles (10,000 vehicles), and the annual grain planting area (10,000 mu). The spatial distribution of the regression coefficients of these factors is shown in Figure 5.

From Figure 5A, it can be seen that the annual grain planting area was negatively correlated with the PM2.5 concentration in northern Shanxi, the northern Beijing–Tianjin–Hebei region, eastern Shandong, and most of Jiangsu and Shanghai, and the rest was positively correlated. The correlation coefficients in northern Shanxi, Zhangjiakou, and Beijing were the smallest, and the correlation coefficients in southern Shaanxi and western Hubei were higher, showing an overall increase from the east to the west. The grain planting area in economically developed regions is relatively small. For example, as the political and economic center of the country, Beijing has developed rapidly. The planting area reserved for grain is relatively small, and the correlation coefficient was relatively small. The annual grain planting area was negatively correlated with the PM2.5 concentration, and the air pollution situation was still severe. In southern Shaanxi, for example, the annual grain planting area in Hanzhong in 2018 was 3.8097 million mu, and the grain planting area was relatively large. Straw burning occurred from time to time, which would cause serious air pollution. The annual grain planting area was positively correlated with PM2.5 concentration.

Figure 5B shows that in cities except for Jiangsu, southeast Anhui, and Shanghai, the number of civilian vehicles was positively correlated with PM2.5 concentration, and increased from the southeast to the northwest. Traffic is one of the major factors affecting air pollution. The larger the number of civilian vehicles, the more serious the pollution is. There was a relatively strong correlation between the ownership of civilian vehicles and PM2.5 concentration in most areas of Shaanxi Province. For example, Xi'an has carried out special rectification actions for motor vehicle exhaust pollution in recent years. Since 2016, the concentration of PM2.5 in Xi'an has decreased significantly in winter, and the effect of pollution control and haze reduction was obvious. However, heavy haze weather still occurs. The contribution rate of heavy trucks to PM2.5 pollution in Xi'an was 47.0%, and that of diesel vehicles was 80.2% (46). Therefore, Xi'an should focus on controlling heavy trucks and diesel vehicles to form a targeted management pattern.

As can be seen from Figure 5C, the urban construction land area was positively correlated in the west of the hotspot area and was negatively correlated in the rest of the area. Shaanxi and western Shanxi had the strongest positive correlation, indicating that the larger the urban construction land area, the higher the annual average PM2.5 concentration was. For example, Yulin is located in the Loess Plateau and is carrying out urban construction in a large area. The dust generated by a large number of construction projects will cause serious air pollution. The soil of the Loess Plateau is thick and loose, which aggravates the increase in PM2.5 concentration.

According to Figure 5D, the proportion of the secondary industry in the GDP was significantly positively correlated with PM2.5 concentration; it decreased from the west to the east, and the correlation coefficient was the highest in eastern Shandong. The second industry is a major force in environmental pollution. The higher the proportion of the second industry in the GDP, the more serious the air pollution is. Yantai is a traditional, strong industrial city, and its industrial output value ranks first in Shandong Province. Yantai vigorously develops the secondary industry, so that the pollution emissions are serious. Yantai should change its urban economic structure, reduce the proportion of the secondary industry, avoid the excessive agglomeration of the same type of secondary industry in the region, and improve the current situation of environmental pollution.

It can be seen from Figure 5E that the green area was negatively correlated with PM2.5 pollution. The high-value area was located in Shanxi and northwestern Shaanxi, and the low-value area was located in Enshi, Chengde, and Qinhuangdao. The larger the green area was, the more conducive it was to reducing PM2.5 pollution. Yan'an belongs to northern Shaanxi, and the green space area had a relatively obvious negative correlation with PM2.5 concentration. Green space helps to improve air pollution; therefore, green space can be further expanded in cities.

It can be seen from Figure 5F that the annual average population was positively correlated with PM2.5 concentration. The high-value area was located in Anhui, southeast Jiangsu, and Shanghai, and the low-value area was located in southern Shaanxi. The larger the annual population is, the more frequent human activities are, and this leads to more serious pollution and higher PM2.5 concentrations. In Shanghai, with a large population, the annual average population was positively correlated with PM2.5 concentration. It is necessary to promote a green low-carbon lifestyle and advocate for the participation of all people in haze prevention and control. In Shandong, Henan, and other populous provinces, the population should be reasonably controlled, and the population structure should be optimized.

It can be seen from Figure 5G that per capita GDP was negatively correlated with PM2.5 concentration. A region with a high per capita GDP has a higher level of economic development, a reasonable industrial structure, and advanced technology. Therefore, the region is more environmentally friendly and produces less pollution. The high-value area was located in the north-central Shaanxi, and the low-value area was located to the south of the hotspot. The per capita GDP of Suzhou was 173,765 Chinese yuan, and the level of economic development was relatively high. The average annual PM2.5 concentration in 2018 was 42 μg/m^3^. The per capita GDP was negatively correlated with PM2.5 concentration. With the rapid economic development of the city, the government should pay attention to the sustainable development of the environment and reduce air pollution.

It can be seen from the above analysis that the seven factors selected in this study had different effects on the average annual PM2.5 concentration. Comparing the average absolute value of the correlation coefficient, the influence degree of each factor on the average annual PM2.5 concentration decreased in the following order: the proportion of the secondary industry in the GDP, the ownership of civilian vehicles, the annual grain planting area, the annual average population, the urban construction land area, the green space area, and the per capita GDP. The proportion of the secondary industry in the GDP has a great influence on PM2.5 concentration. In some areas, the proportion of the secondary industry in the GDP is high, so pollution emissions are serious. It is necessary to accelerate the transformation of the urban economic structure and increase the proportion of the tertiary industry in the economic structure. At the same time, it is necessary to change the urban economic structure, reduce the proportion of the secondary industry, avoid the excessive aggregation of the same type of secondary industry in the region, and improve the current situation of environmental pollution. In Shaanxi Province, attention should be paid to air pollution in urban construction; strict regulations should be made on the air standards for construction, and a supervision system should be implemented to ensure that air pollution caused by construction is controlled within a reasonable range. At the same time, attention should be paid to air pollution caused by automobile exhaust emission pollution and crop planting. The number of cars should be further controlled, green travel should be promoted, penalties should be strengthened for vehicles with unqualified emissions, supervision should be strengthened in agriculture, and straw burning should be strictly prevented. Good economic conditions are the basis for effective environmental protection, so it is urgent to accelerate the high-quality growth of regional GDP. At the same time, expanding the green space in various regions helps reduce air pollution. Because of the differences in atmospheric resource endowments and the functional positioning of each region and its cities, it is recommended to establish a regional classification management system and ecological compensation strategy.

### Hierarchical Management Policies

**Figure 6:**
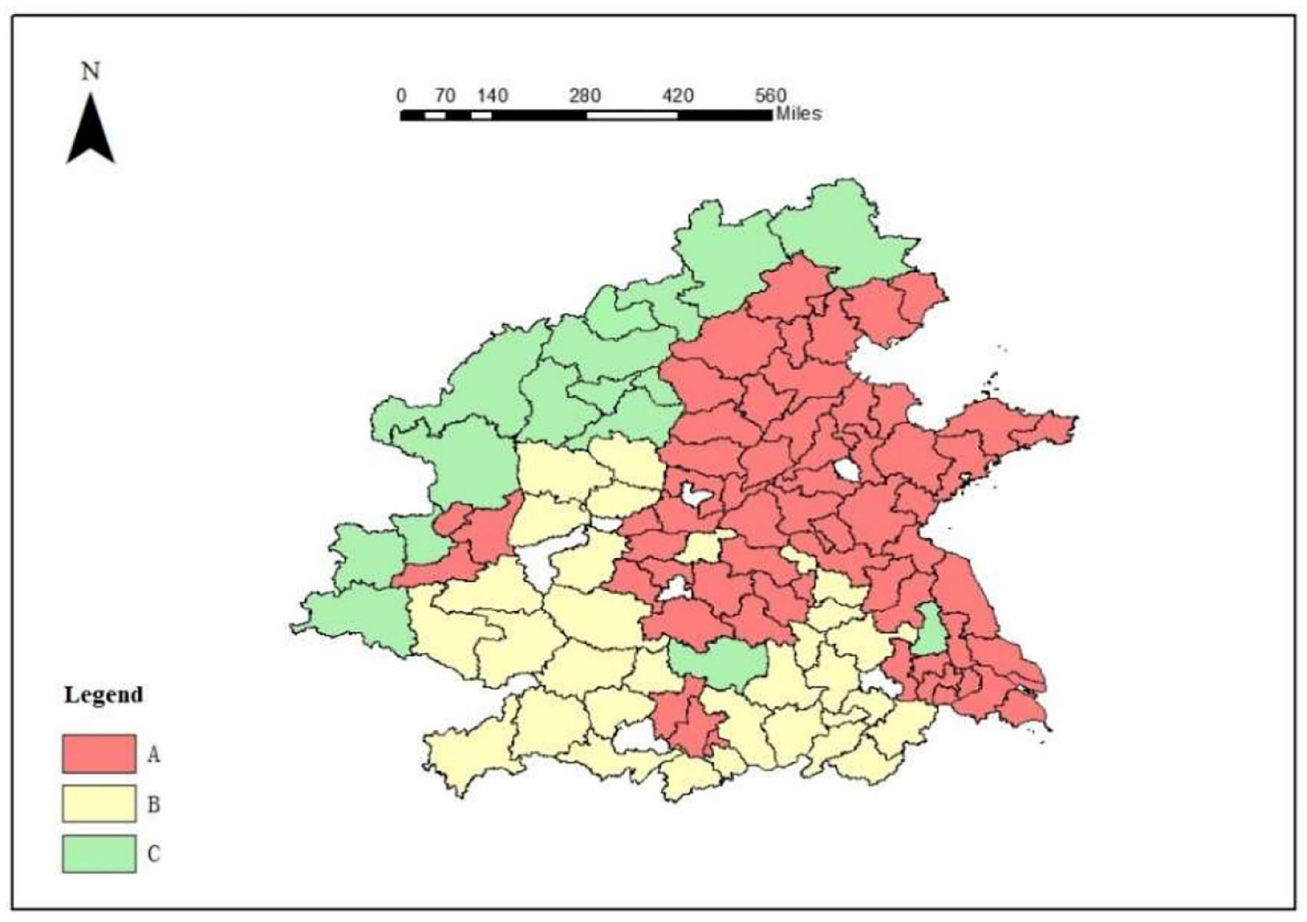
Zoning diagram of the key hotspots.

As mentioned above, PM2.5 pollution has improved year by year from 2018 to 2020 and there was a significant spatial agglomeration phenomenon in the AM distribution of PM2.5 in Chinese cities. According to the local autocorrelation analysis of pollution hotspots, integrated with the population density map (Figure 4A), the key hotspots were divided into three areas: Area A, Area B, and Area C. The classification of key hotspots is shown in Figure 6. According to the analysis of socioeconomic driving factors, we should pay more attention to sustainable development for Area A (63 cities), and the green industry is one of the solutions. For example, Xi'an, Tongchuan, and Weinan are typical cities in Area A, and they are surrounded by the cities in Area B and Area C. To achieve the regional coordinated management of fine particulate pollution and joint economic development, the idle environmental capacity of the surrounding cities can be used to update the joint industrial layout, to optimize the economic and energy structure and further promote the growth of per capita GDP. At the same time, the air standards for urban construction should be strictly stipulated, and the supervision system should be implemented to ensure that the air pollution generated by construction is controlled within a reasonable range. Attention should be paid to the emission pollution of automobile exhaust, green travel should be advocated, and penalties should be strengthened for vehicles that do not meet the emission standards. In the above three cities and Xiaogan, Wuhan, Ezhou, Jiaozuo, Zhengzhou, Zhumadian, Zhoukou, and Xuchang, the government should strictly prevent and control the open-air burning of straw and implement a global full-time comprehensive prohibition of such burning. In Shijiazhuang and Baoding, it is proposed to form a targeted management pattern for heavy trucks and diesel vehicles. In Xuzhou, Lianyungang, Zibo, Linyi, Weihai, Rizhao, Yantai, Weifang, Qingdao, Jining, Dongying, Zaozhuang, Binzhou, and Jinan, governors should pay attention to the air pollution caused by the secondary industry, change the urban economic structure, reduce the proportion of the secondary industry, prevent the same type of secondary industry from becoming too concentrated in the region, and improve the status of environmental pollution. In Shanghai, Taizhou, Nanjing, Changzhou, Yancheng, Zhenjiang, Suzhou, Wuxi, and Nantong, a green and low-carbon lifestyle should be promoted among citizens, and all people should participate in the prevention and control of haze. The cities in Area B were mainly concentrated in southeastern Shaanxi, southwestern Shanxi, western Henan, Anhui, and most parts of Hubei. For the cities in Area B (a total of 30 cities), it is crucial to develop low-emission industries and short-distance tourism to promote economic integration. For example, decision-makers in Shangluo, Ankang, Shiyan, and Enshi should pay attention to air pollution caused by crop planting, strengthen supervision in agriculture, and strictly prevent straw burning. Decision-makers in Linfen, Changzhi, Yuncheng, Shangluo, Ankang, and Enshi should pay attention to automobile exhaust pollution, promote green travel, and focus on the control of heavy trucks and diesel vehicles. In Hefei, Bengbu, Huainan, Chuzhou, Suzhou, Lu'an, and Kaifeng, it is necessary to accelerate the transformation of the urban economic structure, reduce the proportion of the secondary industry and develop low-emission industries. Area C (16 cities in total) was located in Shaanxi, Shanxi, and northern Hebei. To accelerate the growth of the per capita GDP, good economic conditions are the basis for effective environmental protection and contribute to sustainable development. It is necessary to promote the further improvement of the regional cooperation mechanism in key hotspots, establish a comprehensive regional ecological environment management platform and traffic information platform, promote information sharing, implement joint law enforcement and mutual supervision, learn long-term mechanisms, and improve the emergency linkage mechanism to deal with heavy pollution weather, as well as improve the air quality prediction mechanism (47).

At the same time, the seasonal characteristics of PM2.5 pollution were also worthy of attention. Considering that different cities have different roles and different sensitivities to different socioeconomic driving factors, it is urgent to establish clear transfer compensation policies for haze control cooperation between cities in the first and fourth quarters. In addition, the analysis of pollution centers in the four quarters of 2018 showed that the pollution centers were located in the Huanggang, Anqing, Jiujiang, and Shangrao areas, and the government should focus on the coordinated planning and management of the environmental economy in the above areas. Moreover, the air pollution in the heating period in the north was serious. To build a coal-free area and achieve the goal of clean heating, it is necessary to eliminate the high emission of boilers, reform the gas boilers, strengthen the source management, and strictly control the amount of coal used.

## Conclusion

PM2.5 pollution has improved year by year from 2018 to 2020. There was a significant spatial agglomeration phenomenon in the AM distribution of PM2.5 in Chinese cities. Based on the local autocorrelation analysis of pollution hotspots and population density features, the Beijing–Tianjin–Hebei region, the Fenwei Plain, the Yangtze River Delta, and surrounding areas were selected as the key hotspots for GWR analysis. The influence degree of each socioeconomic determinant on the average annual PM2.5 concentration decreased in the following order: the proportion of the secondary industry in the GDP, the ownership of civilian vehicles, the annual grain planting area, the annual average population, the urban construction land area, the green space area, and the per capita GDP. Based on the local autocorrelation analysis of pollution hotspots, when integrated with the population density map, the key hotspots were divided into three areas: Area A (63 cities), Area B (30 cities), and Area C (16 cities). Finally, the targeted management policies of each hierarchical area were proposed according to the characteristics of the driving forces of sensitive pollution in each city as well as the roles of each city, combined with the seasonal characteristics of pollution. The cities in Area A should update the joint industrial layout with the surrounding cities to optimize their economic and energy structure. Cities in Area B should consider transitioning to eco-tourism, eco-agriculture, and other green industries to maintain people's livelihoods. Cities in Area C should accelerate the growth of the per capita regional GDP. Cities in Area D should implement more afforestation to increase the coverage of green plants, which would improve pollution. Moreover, the quarterly pollution focus should be on the coordinated planning and management of the environment and economy in the above-mentioned areas. At the same time, air pollution was serious during the heating season in the north, so the government should strengthen source management and strictly control the amount of coal used.

## Data Availability Statement

The raw data supporting the conclusions of this article will be made available by the authors, without undue reservation.

## Author Contributions

MZ: writing—original draft preparation and data analysis. JG and YZ: data analysis. XC: methodology, reviewing, and revision. All authors contributed to the article and approved the submitted version.

## Funding

This study was supported by the Humanities and Social Science Research Youth Project funded by the Ministry of Education of China (18YJCZH021), Fundamental Research Funds for the Central Universities from Zhongnan University of Economics and Law (202211412 and 202211413).

## Conflict of Interest

The authors declare that the research was conducted in the absence of any commercial or financial relationships that could be construed as a potential conflict of interest.

## Publisher's Note

All claims expressed in this article are solely those of the authors and do not necessarily represent those of their affiliated organizations, or those of the publisher, the editors and the reviewers. Any product that may be evaluated in this article, or claim that may be made by its manufacturer, is not guaranteed or endorsed by the publisher.
