# Supplementary material for: Exploring the Spatiotemporal Evolution and Socioeconomic Determinants of PM2.5 Distribution and Its Hierarchical Management Policies in 366 Chinese Cities
Source: Front Public Health. 2022 Mar 9;10:843862. doi: 10.3389/fpubh.2022.843862 (PMC8959385; doi:10.3389/fpubh.2022.843862)
Supplement: Supplementary file 1 [file Data_Sheet_1.docx]

##### Spatial autocorrelation analysis method

(a) Global autocorrelation

Global autocorrelation uses a single value to describe the correlation of the entire study area. The research methods of global spatial autocorrelation mainly include Moran's I, Geary's G and so on. In this study, Global Moran's I method *is* used for global autocorrelation analysis. The formula is as follows:

 (S1)

 (S2)

where *n* is the number of air quality monitoring points which meet the statistical validity of the data; *x_i_* and *x_j_* represent observation values; *w_ij_* is a spatial weight matrix generated by GeoDa software based on Euclidean distance weight. The range of global Moran’s I is [1, 1], and the value less than (greater than) 0 indicates negative (positive) correlation. The smaller (larger) the value is, the stronger the spatial dispersion (aggregation) of the target value is. When the Moran’s I is close to or equal to 0, it shows that there is a random distribution or no correlation between the observed values. The *Z* value of standardized statistics is usually used to test the spatial autocorrelation of the visibility, calculated as follows:

$Z=\frac{I-E(I)}{\sqrt{VAR(I)}}$ (S3)

where *E (I)* and *VAR (I)* represent the expectation and variance of Moran’s I, respectively.

(b) Local autocorrelation

Global spatial autocorrelation can only determine whether the geographical variables have spatial autocorrelation and reflect the correlation strength; it cannot reflect the characteristics of urban spatial agglomeration in a region. Therefore, the local Moran’s I is used to determine the local spatial autocorrelation of atmospheric PM2.5 pollution and identify its distribution and aggregation patterns. The local Moran’s I is calculated as follows:

 (S4)

where the meaning of *x*, *n*, and *w_ij_* is shown in Formula (S1). The local Moran’s I can also be measured by *Z(I)*. At the confidence level of 0.05, if *Z* ≥ 1.96, cities with high (low) PM2.5 concentrations are surrounded by cities with high (low) PM2.5 concentrations, indicating high- high (low-low) spatial clustering; if *Z* ≤ -1.96, cities with high (low) PM2.5 concentration are surrounded by cities with low (high) PM2.5 concentration, indicating high-low (low-high) spatial clustering. When *Z* = 0, the observation results show an independent random distribution. When -1.96 < *Z* < 1.96, it indicates that the spatial correlation of observed values is not significant.

Getis-Ord* is a cluster analysis tool that identifies the statistical distinct spatial clustering of high (HSs) and low (CSs) values. Gi* is calculated as follows[^[[1]](#endnote-1)^]:

 (S5)

where *Z* values and p values are measures of statistical significance. The larger (or smaller) the *Z* value is, the stronger the clustering of high (or low) values are. The *Z* value close to zero indicates no obvious spatial clustering.

##### Gravity model analysis

From 2018 to 2019, the center of gravity offset distance was farther than that from 2019 to 2020, but the offset angle was small. The reason for the overall stability of the pollution center of gravity was that the natural conditions such as urban location, ocean currents, topography, economic structure and population distribution have not changed much. From 2018 to 2020, the national PM2.5 pollution status was generally at a good level, which was inseparable from the introduction of relevant atmospheric protection policies. In June 2018, the "*Three-year Action Plan to Fight Air Pollution*" was proposed. Based on the overall improvement of air quality, the Beijing-Tianjin-Hebei region and its surrounding areas and several key areas of the Yangtze River Delta were the main battlefields. Through the three-year atmospheric prevention and control work from 2018 to 2020, the main goal of significantly reducing the concentration of fine particulate matter PM2.5 and significantly reducing the number of days of heavy pollution was further achieved, meeting the expectations of the masses and the requirements of high-quality development. Overall, the spatial variation of PM2.5 barycenter was not significant, which indicated that the national PM2.5 pollution pattern was generally stable, in line with the overall pattern of stability, micro-pattern fluctuations.

##### Table S1 PM2.5 concentration standards in *Ambient air quality standards* (GB3095-2012)

|  | limiting values/μg/m³ | |
| --- | --- | --- |
|  | Level I | Level II |
| daily average of PM2.5 concentrations | 15 | 35 |
| hourly average of PM2.5 concentrations | 35 | 75 |

##### Table S2 The number of Chinese cities in different PM2.5 concentration intervals

| PM2.5 concentration/μg/m³ | 15-25 | 25-35 | 35-55 | 55-75 | >75 |
| --- | --- | --- | --- | --- | --- |
| 2018 | 0 | 34 | 222 | 103 | 7 |
| 2019 | 2 | 86 | 216 | 61 | 1 |
| 2020 | 4 | 99 | 247 | 14 | 2 |

##### Table S3 Spatial autocorrelation analysis results

| Items | Values |
| --- | --- |
| Global Moran's I | 2.304041 |
| Variance | 0.000971 |
| Z value | 73.941729 |
| P value | 0.000000 |

##### Fig. S1 The number of Chinese cities in different PM2.5 concentration intervals


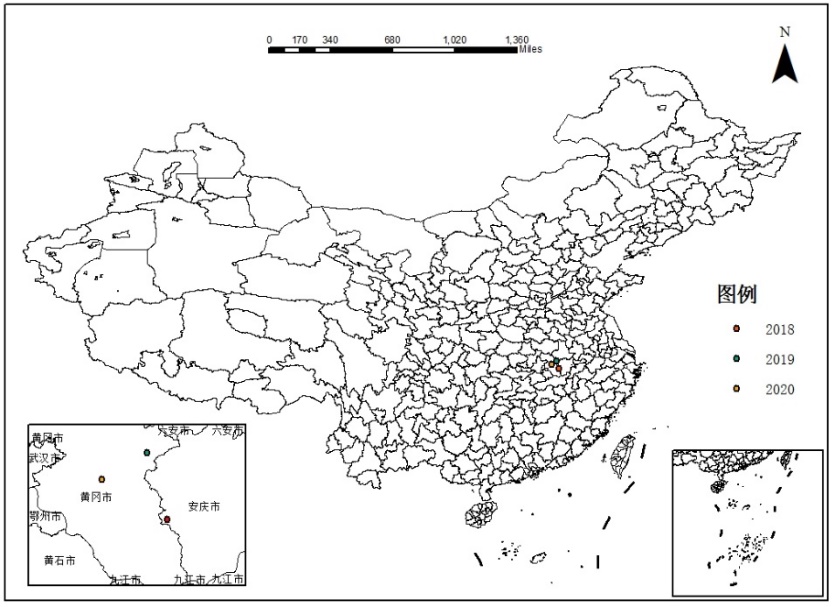


##### Fig. S2 Distribution map of pollution gravity center in 2018-2020


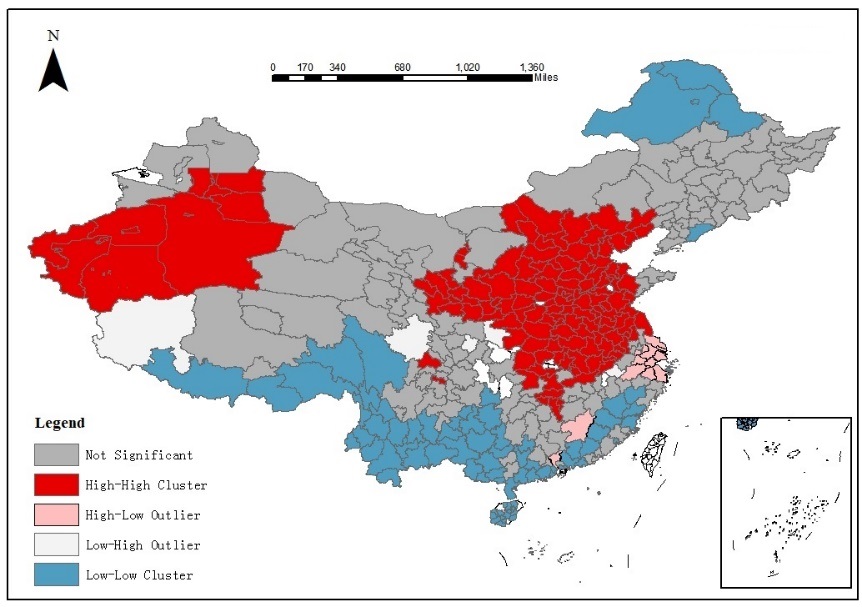


##### Fig. S3 Local Moran’s I

1. [1] Fang, C, Wang, Z., and Xu, G. (2016). Spatial-temporal characteristics of PM2.5 in China: a city-level perspective analysis. *Journal of Geographical Sciences* 26(11), 1519-1532. doi: 10.1007/s11442-016-1341-9. [↑](#endnote-ref-1)
